# Choosing a Cluster Sampling Design for Lot Quality Assurance Sampling Surveys

**DOI:** 10.1371/journal.pone.0129564

**Published:** 2015-06-30

**Authors:** Lauren Hund, Edward J. Bedrick, Marcello Pagano

**Affiliations:** 1 Department of Family and Community Medicine, University of New Mexico, Albuquerque, NM, USA; 2 Department of Biostatistics and Informatics, University of Colorado, Aurora, CO, USA; 3 Department of Biostatistics, Harvard School of Public Health, Boston, MA, USA; Liverpool School of Tropical Medicine, UNITED KINGDOM

## Abstract

Lot quality assurance sampling (LQAS) surveys are commonly used for monitoring and evaluation in resource-limited settings. Recently several methods have been proposed to combine LQAS with cluster sampling for more timely and cost-effective data collection. For some of these methods, the standard binomial model can be used for constructing decision rules as the clustering can be ignored. For other designs, considered here, clustering is accommodated in the design phase. In this paper, we compare these latter cluster LQAS methodologies and provide recommendations for choosing a cluster LQAS design. We compare technical differences in the three methods and determine situations in which the choice of method results in a substantively different design. We consider two different aspects of the methods: the distributional assumptions and the clustering parameterization. Further, we provide software tools for implementing each method and clarify misconceptions about these designs in the literature. We illustrate the differences in these methods using vaccination and nutrition cluster LQAS surveys as example designs. The cluster methods are not sensitive to the distributional assumptions but can result in substantially different designs (sample sizes) depending on the clustering parameterization. However, none of the clustering parameterizations used in the existing methods appears to be consistent with the observed data, and, consequently, choice between the cluster LQAS methods is not straightforward. Further research should attempt to characterize clustering patterns in specific applications and provide suggestions for best-practice cluster LQAS designs on a setting-specific basis.

## Introduction

When designing global health monitoring and evaluation (m&e) surveys in resource limited settings, the key objective is to balance cost, precision, and feasibility. Lot quality assurance sampling (lqas) surveys were originally proposed for industrial quality control [1] and have more recently been applied in global health m&e for classification of health indicators [2]. In lqas surveys, samples of size *n* are collected from supervision areas (sas), and the number of successes *X* is compared to a decision rule *d* to classify an sa as acceptable or unacceptable [3]. Because lqas is a classification procedure, lqas surveys typically require smaller sample sizes than estimation-based surveys. Further, the simplicity of classification facilitates local-level data analysis, reducing the time between data collection and data-driven actions.

Lqas designs are typically determined by four parameters *p*_*l*_, *p*_*u*_, *α*, and *β*. Specifically, the sample size *n* and rule *d* are selected such that the risk of misclassifying an sa as acceptable or unacceptable is small, namely:

$$\begin{array}{ccc} P(X\leq d|p\geq p_{u}) & \leq & \alpha \\ P(X>d|p\leq p_{l}) & \leq & \beta . \end{array}$$ (1)

The risk *α* is the probability of classifying as low when *p* = *p*_*u*_, i.e. coverage is truly high; and the risk *β* is the probability of classifying as high when the health indicator coverage *p* = *p*_*l*_, i.e. coverage is truly low. The risk *α* can then be interpreted as the ‘service provider risk,’ namely the risk of intervening in an area that actually has high coverage; and the risk *β* interpreted as the ‘population risk,’ namely the risk that a truly under-performing area is classified as having adequate coverage. It is important to note that the lqas equations in 1 are often parameterized differently in the literature depending on the application, and the interpretation of the risks changes with the parameterization. The *α* and *β* risks are not restricted for sas with *p*_*l*_ < *p* < *p*_*u*_, i.e. in the “grey region.” Choice of the design parameters *p*_*l*_, *p*_*u*_, *α*, and *β* has been discussed elsewhere [3–5] and is not addressed in this paper.

Typically, the probabilities in Eq 1 are calculated by assuming data are collected using simple random sampling (srs) from the target population and then modeling *X* ∼ *Binomial*(*n*, *p*). Implementing srs requires enumerating and randomly sampling individuals from the target population, a challenging feat in geographically disperse and hard to reach populations. Cluster sampling surveys are popular in m&e, where enumerating clusters (often villages) and sampling within clusters is less costly [6]. Commonly used rapid m&e cluster surveys include 30 × 30 emergency nutrition surveys [7, 8] and 30 × 7 Expanded Program of Immunization (epi) surveys [9].

Recently, three methods have been proposed to adjust lqas sample sizes and decision rules to accommodate cluster sampling. We refer to these methods as the Pezzoli [10–13], Hedt [14], and Hund [15] methods. In this paper, we contrast these methods and provide recommendations for choosing a cluster lqas design. First, we review two-stage cluster sampling surveys and describe the three methods for melding cluster sampling with lqas. We then evaluate these methods using example designs from vaccination and nutrition surveys. Finally, we provide recommendations for choosing a cluster lqas design based on these results.

## Methods

### Cluster surveys

Global health m&e surveys often implement two-stage cluster sampling, with clusters sampled using probability proportional to size (pps) and individuals sampled using an approximation to srs. When clusters are sampled with replacement (or the probability of sampling the same cluster more than once is small), this design is ‘self-weighting’. That is, every individual in the target population has an equal probability of being sampled and the data do not have to be weighted during the analysis. Cluster lqas decision rules rely on use of self-weighting designs, and throughout this paper we assume that data are collected using a self-weighted two-stage cluster sampling design.

When individuals within a cluster are more similar than those between clusters, the data cannot be analyzed as a simple random sample. Consider a population of size *N* consisting of *K* total clusters of size *M*_*j*_ for cluster *j*. Define *X*_*ij*_ as the health indicator status (1 success, 0 failure) of subject *i* in cluster *j*; *X*_*j*_ = ∑_*i*_ *X*_*ij*_ as the number of ‘successes’ in cluster *j*; and *X* = ∑_*j*_ *X*_*j*_ as the number of ‘successes’ in the sample. Suppose *k* clusters are sampled and *m* individuals are sampled per cluster (*n* = *km*) and define *p*_*j*_ as the coverage in cluster *j*. In the presence of clustering, the cluster-level coverages {*p*_*j*_} differ from the overall coverage *p*; one measure of clustering is the standard deviation of *p*_*j*_ across clusters, denoted *σ*. Another measure of clustering is the intraclass correlation, *ρ*. For two-stage designs in which cluster coverages {*p*_*j*_} have mean *p* and standard deviation *σ* and *X*_*j*_∣*p*_*j*_ ∼ *Binomial*(*m*, *p*_*j*_), the intraclass correlation is defined as [16]:

$$\begin{array}{c} \rho =\frac{\sigma^{2}}{p(1-p)}. \end{array}$$ (2)

The intraclass correlation *ρ* varies between 0 and 1 with higher values denoting more clustering (technically, under alternative models, *ρ* can be negative when individuals within a cluster are less similar than between clusters, though this setting is typically not relevant to m&e surveys and is not considered here).

### Cluster sampling in LQAS

Initially, cluster sampling lqas surveys were designed to minimize the amount of variance inflation due to clustering without directly inflating the sample size for clustering. For instance, within-cluster sample sizes of *m* = 1 have been used in lqas surveys (*e.g*. [17]; [18]) to achieve the same precision as a srs survey. In 67 × 3 and 33 × 6 lqas nutrition surveys, small within-cluster sample sizes were selected to minimize cluster sampling variance inflation [19–21]. Stroh and Birmingham (2009) use cluster sampling for lqas neonatal tetanus elimination surveys and ignore the cluster sampling design, noting that cluster sampling approximates srs ‘when an attribute to be measured in a population is rare or is homogeneously distributed’ [22]. The common theme between these approaches is that the cluster sampling design can be ignored if the within cluster sample size is small or if clustering is negligible. In these situations, the lqas sample size and decision rule can be calculated using the standard binomial model with no adjustments required to accommodate the use of cluster sampling.

In practice, clustering is often not negligible, and visiting many clusters and selecting few individuals per cluster may be less logistically feasible or cost-effective than sampling more individuals per cluster. To address these limitations, three methods have recently been developed to explicitly accommodate clustering sampling at the design-phase in cluster lqas surveys: the Pezzoli [10–13], Hedt [14], and Hund [15] methods. In this paper, we compare these three methods (Hund, Hedt, and Pezzoli) for explicitly accounting for the cluster sampling design and do not discuss designs that ignore the cluster sampling design. We describe these methods below.

#### Pezzoli method

Pezzoli *et. al* [10] proposed the first cluster lqas method, developed to assess vaccination coverage. The mathematical representation of the Pezzoli model described in [11, 12] is:

$$\begin{array}{ccc} p_{j} & ∼ & Binomial(\eta ,p)/\eta \\ sd(p_{j}) & = & \sigma ,\;E(p_{j})=p\\ X_{j} & ∼ & Binomial(m,p_{j}). \end{array}$$ (3)

We refer to this model as a binomial-scaled model, because *p*_*j*_ is modeled as a binomial random variable scaled by *η* to ensure that *p*_*j*_ ∈ (0,1). Clustering is incorporated through specifying a value for *σ*, the standard deviation of the cluster level coverages. To apply the Pezzoli design, *η* is selected such that *σ* ^2^ = *p*(1 − *p*)/*η*. Using the relationship between *σ* and *ρ*, it follows that *η* = 1/*ρ*. This method is subject to some rounding error, since *η* is rounded to an integer.

To select designs, the authors choose *p* = {*p*_*l*_, *p*_*u*_} and *σ* ∈ (0,.1) and determine decision rules via simulation based on the distribution of *X* = ∑_*j*_ *X*_*j*_. There is currently no user-friendly software for implementing the Pezzoli method that allows users to select their own design, though Stata simulation code is available from the authors of [11]. We have written R functions for designing these surveys and provide examples using this code at https://github.com/lbhund/ClusterLQAS_PLOS.

#### Hedt method

Hedt *et. al* [14] propose modeling *X*_*j*_ as a beta-binomial random variable with mean *p* and intraclass correlation *ρ*:

$$\begin{array}{c} X_{j}∼Betabinomial\left(m,p,\rho \right) \end{array}$$ (4)

The betabinomial model can also be written as a two-stage model, with *X*_*j*_ ∼ *Binomial*(*m*, *p*_*j*_) and *p*_*j*_ ∼ *Beta*(*p*, *ρ*) (where the beta distribution is parameterized based on the mean *p* and intraclass correlation *ρ*). The beta distribution has support on (0,1), and this betabinomial model is common for clustered binary data. The final decision rule is based on the distribution of *X* = ∑_*j*_ *X*_*j*_. Hedt *et. al* [14] provide R code for selecting sample sizes and decision rules for this method; the R package in [15] can also be used to calculate sample sizes and decision rules using the Hedt method.

#### Hund method

Hund and Pagano (2014) do not specify a distribution for *p*_*j*_ and instead use an overdispersed binomial model for *X* [15]:

$$\begin{array}{ccc} X & ∼ & Quasibinomial(n,p)\\ E(X) & = & np,\;Var(X)=Dnp(1-p) \end{array}$$ (5)

where *D* is the survey design effect allowing variability in *X* above that of the binomial distribution. The design effect is the ratio of the variances using cluster sampling compared to srs and is the multiplicative factor by which the sample size should be inflated to achieve the same precision as srs when using cluster sampling [23]. For the two-stage cluster sample with large *K*, the design effect is *D* = 1 + (*m* − 1)*ρ*.

The Hund method can also be written in terms of the ‘effective sample size’ as X* ∼ *Binomial*(n*, *p*), where X* = *X*/*D* and n* = *n*/*D* is the effective sample size. Thus, the sample size and decision rule can be calculated under srs and inflated by the design effect to accommodate clustering. The R package in [15] can be used to calculate sample sizes and decision rules for this method. A limitation of the Hund method is that *α* and *β* errors are inexact due to rounding error when transforming from the effective sample size scale.

### Evaluating the designs

There are two primary differences between the methods above: the distributional assumptions (binomial-scaled, beta, and quasi-binomial models) and clustering parameterization using *σ* versus *ρ* (that is, the difference between treating *σ* or *ρ* as known and the same for *p*_*l*_ and *p*_*u*_). These differences are summarized in Table 1. We compare the impacts of these differences across the Pezzoli, Hedt, and Hund methods and determine situations in which the choice of method results in a substantively different design. As example designs, we use three couplets for *p*_*l*_ and *p*_*u*_: 55–70%; 75–90%; and 90–95%. The first two couplets are from the 6 × 10 two-stage cluster designs for vaccination coverage proposed in [12]. The last couplet is consistent with the 5–10% couplet used in the 33 × 6 or 67 × 3 malnutrition LQAS surveys. We parameterize these designs according to Eq 1 and specify *X* as the number of successes (vaccinated children or children with no evidence of malnutrition) out of *n*. As noted above, both the Pezzoli and Hund models are subject to some rounding error; we use nearest integer rounding for all calculations. All analyses have been conducted using R 3.1.1 [24], and code for reproducing all figures and tables, along with the R package from [15], is available at https://github.com/lbhund/ClusterLQAS_PLOS.

**Table 1:** Differences between the 3 methods.

Method	Distribution for *p*_*j*_	Clustering parameterization
Pezzoli	Binomial-scaled	*σ* fixed
Hedt	Beta	*ρ* fixed
Hund	None specified	*ρ* fixed

#### Comparing the distributional assumptions

First, we conduct a simulation study to evaluate whether the choice of distributional model (binomial-scaled, beta, and quasi-binomial) makes a substantive difference in the classification precision of the cluster lqas designs. For the 2 *p*_*l*_ and *p*_*u*_ threshold couplets from [12], we use the 6 × 10 design with decision rules *d* = 38 and 50; for the 90–95% couplet, we use the 33 × 6 design with *d* = 13. We fix *σ* = .1 for all models (we repeat the analysis fixing *σ* = .05; results are similar and are not presented). That is, we select the clustering parameters at *p*_*l*_ and *p*_*u*_ such that *σ* = .1 for all models and thresholds (deviating from the specific methods described above) to isolate the impact of the probability distribution choice. For each *p*_*l*_, *p*_*u*_ couplet, we generate 10,000 draws from each of the three distributions fixing *m*, *k*, *d*, and *σ* = .1 and examine how *α* and *β* change with the distributional assumption. For the quasi-binomial and betabinomial models, we calculate *ρ* using Eq 2. We then repeat the simulation fixing *ρ* = .1 (rather than fixing *σ* = .1) and calculate *σ* for the binomial-scaled model using Eq 2.

#### Fixing *σ* versus *ρ*

Historically, survey sample size calculations rely on the design effect and thus assume *ρ* is known and constant across levels of coverage *p* [16, 25]. The Hund and Hedt methods also model clustering assuming *ρ* is known and fixed as a function of *p*. The Pezzoli method parameterizes clustering differently, assuming *σ* is fixed as a function of *p*. Fixing *ρ* results in less cluster-level variance (smaller *σ*) as *p* moves away from .5. Fixing *σ* results in the same variance of *p*_*j*_ for all levels of *p* (and larger *ρ* as moves away from .5). Intuitively, variability in *p*_*j*_ may decrease as *p* moves away from .5 (e.g. less variability in the coverage of a rare event), though there is also evidence that *ρ* decreases as *p* moves away from .5 [25, 26]. It is thus not immediately clear as to whether fixing *σ*, *ρ*, or neither is appropriate.

To examine design differences according to the clustering parameterization, we compare the Pezzoli, Hund, and Hedt designs when *σ* = .1 and when *ρ* = .1. That is, we first calculate *ρ*_*l*_ and *ρ*_*u*_ such that *σ* = .1 at *p*_*l*_ and *p*_*u*_, respectively, and compare fixing both *ρ*_*l*_ and *ρ*_*u*_ for the Hedt and Hund designs to the Pezzoli design with *σ* = .1. We then repeat this exercise, calculating *σ*_*l*_ and *σ*_*u*_ such that *ρ* = .1 at *p*_*l*_ and *p*_*u*_, respectively, and compare fixing both *σ*_*l*_ and *σ*_*u*_ for the Pezzoli design to the Hedt and Hund designs with *ρ* = .1. We again evaluate the three couplets 55–70%, 75–90%, and 90–95% and specify *α* = *β* = .1 and *m* = 10.

We then examine reported estimates of *p*, *σ*, and *ρ* from [12] and [27] to assess how estimates of *ρ* and *σ* vary as a function of estimated vaccination coverage in practice. Before delving into this data example, we note that the standard deviation of the *estimated* ${\hat{p}}_{j}$ s, $s=\sqrt{{\left(k-1\right)}^{-1}\sum_{j}{\left({\hat{p}}_{j}-\hat{p}\right)}^{2}}$ where $\hat{p}$ is the estimated coverage, is an overestimate of *σ*. Some have criticized the Pezzoli method for using an upper bound of *σ* = .1 in the designs and cite examples of *σ* >.1 in practice. Using *s* as an estimate of *σ* with small *m* likely accounts for some of this phenomenon. While *s* estimates $sd({\hat{p}}_{j})$, $sd({\hat{p}}_{j})$ is higher than *σ*, because $sd({\hat{p}}_{j})$ includes additional variability associated with the estimation of ${\hat{p}}_{j}$. That is, the standard deviation of the *estimated* cluster coverages is not an accurate estimate of *σ*. In the Appendix, we derive an estimator for *σ* ^2^, denoted ${\hat{\sigma }}^{2}$:

$$\begin{array}{ccc} {\hat{\sigma }}^{2} & = & \frac{ms^{2}-\hat{p}\left(1-\hat{p}\right)}{m-1}. \end{array}$$ (6)

From Eq 6, it is clear that using *s* to estimate *σ* is only appropriate when *m* is large (which is seldom the case in cluster lqas surveys).

We estimate $\hat{\sigma }$ using reported $\hat{p}$ and standard errors $s/\sqrt{k}$ for 20 different 6 × 10 surveys in [12]. Minetti *et. al* [27] report $\hat{p}$, *s*, and intraclass correlation estimates $\hat{\rho }$ from 41 different 10 × 15 vaccination coverage surveys. Rather than using Eq 6, we estimate $\hat{\sigma }=\hat{\rho }\hat{p}(1-\hat{p})$ for the data in [27]. For each dataset, we estimate the relationship between $\hat{\rho }$ and $\hat{p}$ and between $\hat{\sigma }$ and $\hat{p}$ using a nonparametric loess smooth [28] based on the loess function in R. We use the default parameters in the loess function, using a weighted least squares fit based on 75% of the data for the local smoothing.

For each data example, we then estimate operating characteristic (oc) curves for the 6 × 10 design with *d* = 50 for the 75–90% couplet using the binomial-scaled distribution but varying the values of *ρ* and *σ*. First, we assume *ρ* is fixed at the value corresponding to the mean of $\hat{\rho }$ across all SAs; next, we allow *ρ* to vary as a function of *p*, estimating *ρ* from the nonparametric loess smooth for $\hat{\rho }$. We then repeat for *σ*, resulting in 4 oc curves for each data example.

## Results

### Distributional assumptions

**Fig 1:**
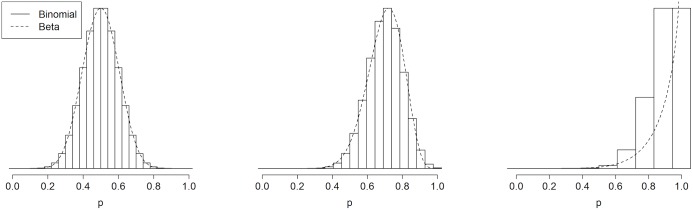
Shape of the beta and binomial-scaled distributions with standard deviation *σ* = .1 and means *p* = .5, *p* = .7, and *p* = .9.

In Fig 1, we contrast the shapes of the beta and binomial-scaled distributions for *p*_*j*_ (the Hund method does not impose a distribution on *p*_*j*_). We plot the shape of these distributions with *σ* = .1 and *p* = .5, .7 and .9. When *p* = .5, these distributions are symmetric and similar. When *p* ≠.5, both distributions are skewed, with the beta distribution more skewed than the binomial. Additionally, as *p* → 1 and as *σ* increases, the binomial distribution becomes more discrete. When *p* = .9 and *σ* = .1, *η* = 9 and *p*_*j*_ can only take on 10 distinct values: (0,1/9,…,8/9,1).

While Fig 1 highlights important differences in beta and binomial-scaled distributions, our simulations suggest that the distribution choice does not have a large impact on the *α* and *β* risk levels (Table 2). Specifically, when the clustering levels are fixed (to either *σ* = .1 or *ρ* = .1), the differences in risks between the binomial-scaled and beta model are negligible for these designs, even with the relatively high levels of clustering specified. The quasi-binomial model generally results in similar *α* and *β* errors to the other models, though differences occur due to rounding error in small sample sizes. Results of these simulations thus suggest that specification of the distribution for *p*_*j*_ does not markedly impact the *α* and *β* errors.

**Table 2:** Comparing distributional assumptions between the methods when *σ* = .1 and *ρ* = .1.

	*p*_*l*_ = .55, *p*_*u*_ = .7		*p*_*l*_ = .75, *p*_*u*_ = .9		*p*_*l*_ = .9, *p*_*u*_ = .95	
	*α*	*β*	*α*	*β*	*α*	*β*
*σ* = .1						
Binomial-scaled	.203	.107	.147	.081	.203	.103
Betabinomial	.195	.109	.143	.088	.203	.107
Quasi-binomial	.159	.160	.072	.169	.204	.103
*ρ* = .1						
Binomial-scaled	.234	.149	.147	.111	.170	.107
Betabinomial	.233	.148	.131	.118	.168	.106
Quasi-binomial	.222	.149	.092	.153	.126	.143

For the 55–70% and 75–90% couplets, the risks for the 6 × 10 design with *d* = 38 and *d* = 50, respectively, are shown. For the 90–95% couplet, the risks for the 33 × 6 design with *d* = 13 are shown. We compare the binomial-scaled (Pezzoli), beta (Hedt), and quasi-binomial (Hund) models.

### Clustering parameterization

In Tables 3 and 4, designs are compared based on the clustering parameterization (fixed *σ* versus fixed *ρ*). For the 55–70% and 75–90% couplets, the designs are similar regardless of how clustering is parameterized. For the 90–95% couplet, the sample size is much larger in the Hedt and Hund methods with fixed *ρ*_*u*_ versus fixed *ρ*_*l*_; similarly, the sample size is much larger in the Pezzoli method with fixed *σ*_*l*_ versus fixed *σ*_*u*_. Recall that the examples in Tables 3 and 4 assume that *m*, the sample size within clusters, is fixed and increments the overall sample size by adding additional clusters. As noted above, clustering variance inflation increases as the within-cluster sample size increases. Subsequently, discrepancies between the three methods are more pronounced when the number of sampled clusters *k* is fixed, rather than m. As an example, if we were to fix the number of clusters to *k* = 6 rather than fixing the within-cluster sample size *m* = 10, the total sample size *n* would range between 72 and 150 when *σ* = .1 (analogous to the 70–90 sample size range in Table 3 with *m* fixed).

**Table 3:** Comparing the Pezzoli, Hund, and Hedt designs for *σ* = .1, *α* = *β* = .1, and *m* = 10.

		n	d	k	m	*α*	*β*
*p*_*l*_ = .55, *p*_*u*_ = .7							
SRS		71	44			.091	.096
Pezzoli	*σ* = .1	100	62	10	10	.090	.096
Hund	*ρ*_*l*_ = .04	110	68	11	10	.069	.091
Hedt	*ρ*_*l*_ = .04	110	68	11	10	.071	.097
Hund	*ρ*_*u*_ = .05	110	69	11	10	.091	.079
Hedt	*ρ*_*u*_ = .05	110	68	11	10	.071	.099
*p*_*l*_ = .75, *p*_*u*_ = .9							
SRS		40	33			.100	.096
Pezzoli	*σ* = .1	80	66	8	10	.088	.076
Hund	*ρ*_*l*_ = .05	70	58	7	10	.093	.071
Hedt	*ρ*_*l*_ = .05	70	58	7	10	.081	.077
Hund	*ρ*_*u*_ = .11	80	66	8	10	.100	.096
Hedt	*ρ*_*u*_ = .11	90	75	9	10	.096	.074
*p*_*l*_ = .9, *p*_*u*_ = .95							
SRS		187	173			.087	.098
Pezzoli	*σ* = .1	440	407	44	10	.095	.091
Hund	*ρ*_*l*_ = .11	380	352	38	10	.096	.087
Hedt	*ρ*_*l*_ = .11	370	343	37	10	.100	.093
Hund	*ρ*_*u*_ = .21	540	501	54	10	.087	.098
Hedt	*ρ*_*u*_ = .21	550	510	55	10	.088	.091

**Table 4:** Comparing the Pezzoli, Hund, and Hedt designs for *ρ* = .1, *α* = *β* = .1, and *m* = 10.

		n	d	k	m	*α*	*β*
*p*_*l*_ = .55, *p*_*u*_ = .7							
SRS		71	44			.091	.096
Pezzoli	*σ*_*l*_ = .16	140	87	14	10	.098	.098
Pezzoli	*σ*_*u*_ = .14	130	81	13	10	.099	.087
Hund	*ρ* = .1	140	87	14	10	.091	.087
Hedt	*ρ* = .1	140	87	14	10	.087	.099
*p*_*l*_ = .75, *p*_*u*_ = .9							
SRS		40	33			.100	.096
Pezzoli	*σ*_*l*_ = .14	110	90	11	10	.063	.095
Pezzoli	*σ*_*u*_ = .09	80	66	8	10	.075	.071
Hund	*ρ* = .1	90	74	9	10	.093	.071
Hedt	*ρ* = .1	90	75	9	10	.088	.073
*p*_*l*_ = .9, *p*_*u*_ = .95							
SRS		187	173			.087	.098
Pezzoli	*σ*_*l*_ = .09	410	379	41	10	.093	.098
Pezzoli	*σ*_*u*_ = .07	320	296	32	10	.088	.094
Hund	*ρ* = .1	360	332	36	10	.093	.090
Hedt	*ρ* = .1	370	343	37	10	.089	.085

**Fig 2:**
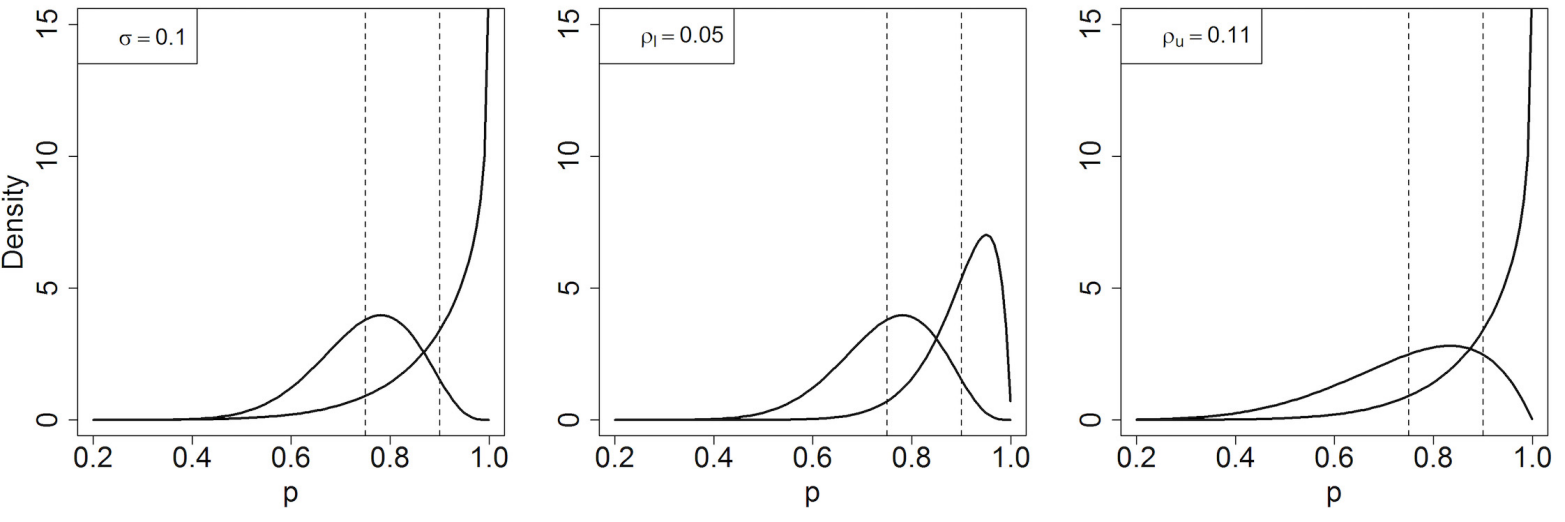
Plot of beta distributions with fixed mean coverages and varying standard deviation and/or intraclass correlation. The dotted lines represent the mean coverages *p*_*l*_ = .75 and *p*_*u*_ = .9, which are the same in all 3 panels. Left: standard deviation is fixed for both distributions at *σ* = .1; middle: intraclass correlation is fixed at *ρ*_*l*_ = .05 (note: *ρ*_*l*_ = .1^2^/(.75*.25)); right: intraclass correlation is fixed at *ρ*_*u*_ = .11 (note: *ρ*_*u*_ = .1^2^/(.9*.1)).

Eq 2 helps explain the sensitivity of the sample size to the clustering parameterization when *p*_*l*_ and *p*_*u*_ are near 0 or 1. The parameters *ρ* and *σ* will be similar for both *p*_*l*_ and *p*_*u*_ when *p*_*l*_(1 − *p*_*l*_) ≈ *p*_*u*_(1 − *p*_*u*_). Hence, *ρ*_*l*_ and *ρ*_*u*_ are negligibly different when *σ* = .1 for the 55–70% couplet, whereas *ρ*_*u*_ is nearly double *ρ*_*l*_ for the 75–90% and 90–95% couplets. To illustrate this difference for the 75–90% couplet, we plot beta distributions fixing *σ* = .1, fixing *ρ*_*l*_, and fixing *ρ*_*u*_ in Fig 2. Unlike the distributional assumptions, choice of clustering parameterization can substantially impact the final sample size for the survey when *p*_*l*_ and *p*_*u*_ are near 0 or 1.

**Fig 3:**
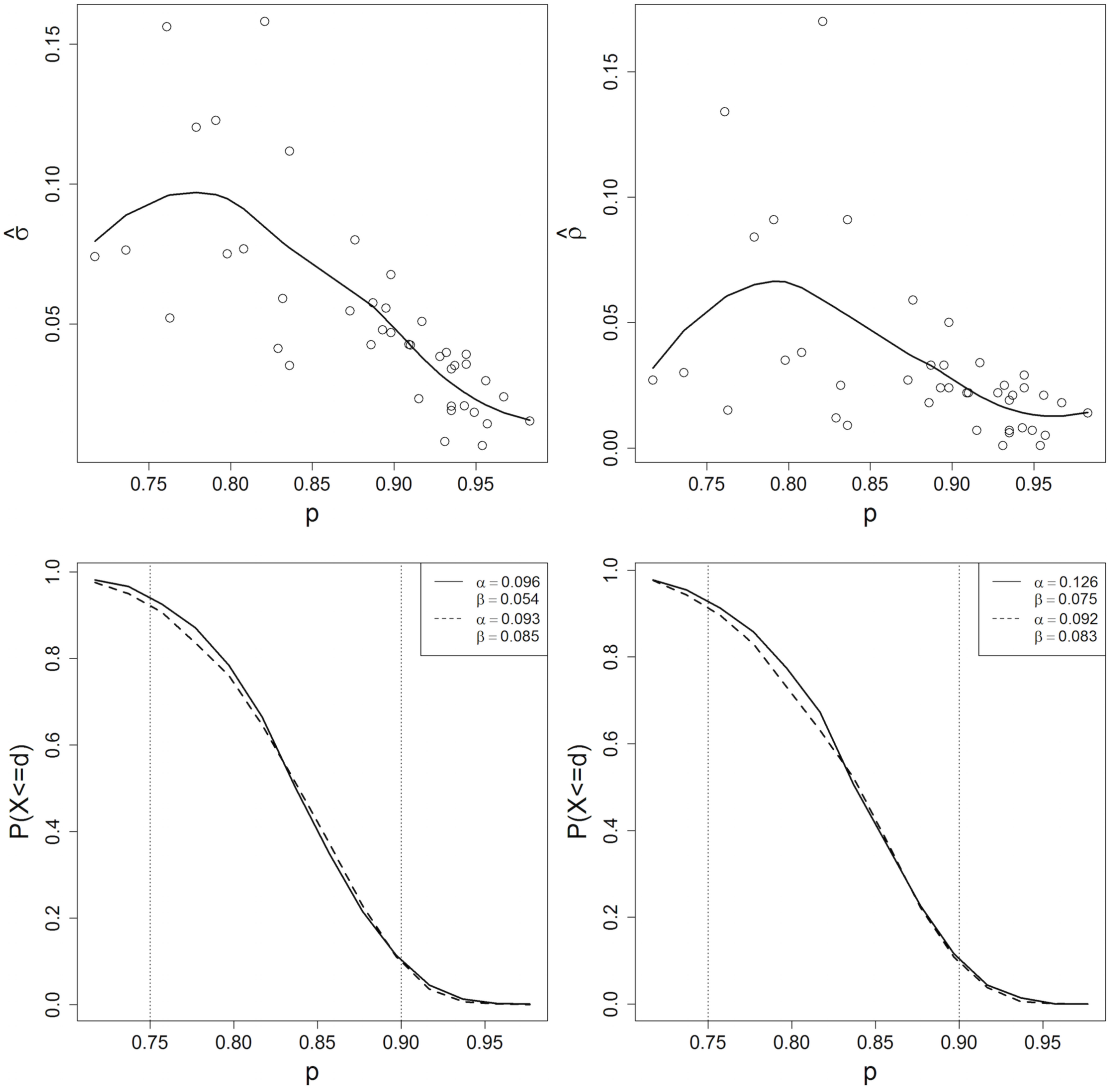
Top panel: Estimated *σ* and *ρ* as a function of ${\hat{p}}_{j}$ for 37 areas in Minetti *et. al* [27], with loess smooth overlayed. Bottom panel: Solid line represents OC curve for *n* = 60, *d* = 50 for *p*_*l*_ = .75, *p*_*u*_ = .9 design when *σ* and *ρ* are fixed at the mean value of $\hat{\sigma }$ (left) and $\hat{\rho }$ (right). Dashed line represents OC curve when *σ* and *ρ* vary over *p* according to the predicted loess smooth of $\hat{\sigma }$ (left) and $\hat{\rho }$ (right).

**Fig 4:**
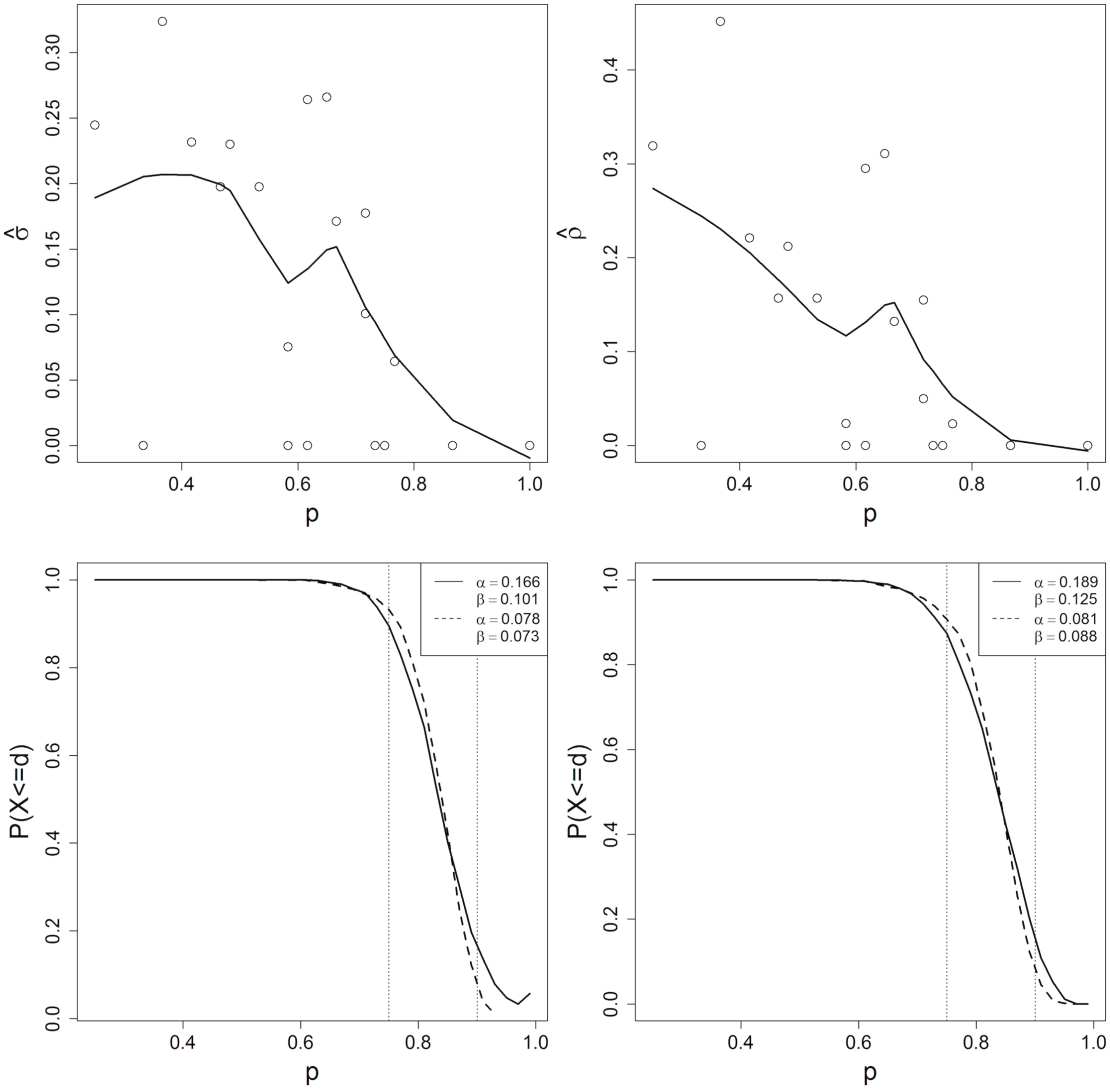
Top panel: Estimated *σ* and *ρ* as a function of ${\hat{p}}_{j}$ for 20 areas in Greenland *et. al* [12], with loess smooth overlayed. Bottom panel: Solid line represents OC curve for *n* = 60, *d* = 50 for *p*_*l*_ = .75, *p*_*u*_ = .9 design when *σ* and *ρ* are fixed at the mean value of $\hat{\sigma }$ (left) and $\hat{\rho }$ (right). Dashed line represents OC curve when *σ* and *ρ* vary over *p* according to the predicted loess smooth of $\hat{\sigma }$ (left) and $\hat{\rho }$ (right).

Next, we examine whether existing data support one of the two proposed parameterizations. In Figs 3 and 4, $\hat{\sigma }$ and $\hat{\rho }$ are plotted as a function of $\hat{p}$ for the data in [12] and [27], respectively. Both $\hat{\rho }$ and $\hat{\sigma }$ decrease as a function of estimated vaccination coverage (less clustering occurs in more highly vaccinated areas), consistent with [25] and [26]. Fixing either *σ* or *ρ* likely results in some level of misspecification of *α* and *β*. The oc curves corresponding to the different clustering specifications are also shown in Figs 3 and 4. In the example from [12], the *α* and *β* risks are substantially higher when the mean versus the lowess smooth estimate of *σ* and *ρ* are used. This inflation of the risks occurs because *σ* and *ρ* decrease as a function of *p* but the average coverage across sas is lower than 75%. The risks are not inflated in the example from [27] because the range of vaccination coverages is similar to the 75–90% couplet thresholds.

## Discussion

After reviewing three methods for implementing cluster sampling in lqas surveys, we conclude that the cluster lqas methods result in similar designs when *p*_*l*_ and *p*_*u*_ are far from 0 or 1 or when clustering is minimal. In these situations, the user should not be concerned about which of the cluster methods to use, as the methods should all result in similar designs. Our simulation study suggests that the choice of distributional assumptions also does not appear to substantively impact the survey design.

Choice of the clustering parameterization is the major factor that can impact the final cluster lqas survey design. Specifically, if *p*_*l*_ or *p*_*u*_ is near 0 or 1, the design (sample size) changes substantially depending on the clustering parameterization, namely whether *ρ* or *σ* is fixed as a function of *p*. That is, when *p*_*l*_ ≤.1 and *p*_*u*_ ≥.9, clustering parameters should be selected carefully to avoid severe misspecification of classification risks. As an example, assume *p*_*l*_, *p*_*u*_ >.5. When *σ* and *ρ* result in the same amount of clustering for *p* = *p*_*l*_ (that is, *σ* = *ρp*_*l*_(1 − *p*_*l*_)), the design with fixed *σ* will require a larger *n*; for *p* = *p*_*u*_, the design with fixed *ρ* will require a larger *n*. Differences in *n* between these options can be substantial when *p*_*u*_ is close to 1 or *p*_*u*_−*p*_*l*_ is large.

We did not find evidence that *ρ* is constant as a function of *p* (assumed by Hedt and Hund) nor that *σ* is constant as a function of *p* (assumed by Pezzoli). In two vaccination coverage examples, estimates of both *ρ* and *σ* decrease as coverage increases, consistent with [25, 26]. Using historical data to plot clustering estimates $\hat{\sigma }$ and $\hat{\rho }$ as a function of estimated coverage $\hat{p}$ can help evaluate the clustering parameterization assumptions. Our data analysis suggests that, if clustering design parameters (*σ* or *ρ*) are determined from historical data, only estimates of $\hat{\sigma }$ and $\hat{\rho }$ from sas with estimated coverages near the range of *p*_*l*_ and *p*_*u*_ should be incorporated. To facilitate incorporation of historical data, we provide r functions that estimate *σ* using summary statistics commonly available in the literature. We strongly encourage cluster lqas users to publish their estimates of these summary statistics ($\hat{p}$, *s*, and $\hat{\rho }$) for each sa to provide more historical data for specifying these design parameters in the future.

When the Hund or Hedt methods are used, we propose the following steps to choose a design:

1. Define *p*_*m*_ as whichever of *p*_*l*_ or *p*_*u*_ is closer to .5. Estimate *ρ* using existing data or existing literature using sas with coverage estimates near *p*_*m*_. If data are not available, consider how much variability is expected around *p*_*m*_ to select *ρ*.
2. Determine the cost of sampling a cluster and sampling each individual per cluster.
3. Iterate through choices of *m* and *k* to minimize cost for *α*, *β*, *p*_*l*_, *p*_*u*_, and *ρ*.

The same steps could be applied to the Pezzoli method, replacing *ρ* with *σ*. The r package accompanying this paper can help achieve Steps 1 and 3. Additionally, users can examine the sensitivity of their designs to the selected model (as in Tables 3 and 4) by calculating different possible designs with varied clustering parameterizations (after selecting the relevant values of *p*_*l*_, *p*_*u*_, *α* and *β*) using the R package. Knowledge of the design’s sensitivity to the clustering parameterization contributes to understanding the robustness of the design.

In Step 1 above, determining *ρ* or *σ* based on *p*_*m*_ has two advantages: (1) *σ* and *ρ* are likely to decrease as *p* moves away from .5, so this clustering estimate is conservative (note that, using this algorithm, fixing *σ* is more conservative than fixing *ρ*); and (2) visualizing a highly skewed distribution in terms of a mean and standard deviation is not straightforward, and the skewness of the beta and binomial-scaled distributions increases as *p* moves away from .5. Returning to Fig 1, the beta and binomial-scaled distributions with mean *p* = .5 are symmetric, and one can easily visually check that *σ* = .1 by examining the spread of the distribution. With mean *p* = .9, there is no way to visually inspect these highly skewed distributions and conclude that *σ* = .1.

If the clustering parameters are underestimated in practice, the survey design will not have the desired precision; that is, the true *α* and *β* risks will be higher than those specified by design. One way to protect against this situation is to employ a sequential design [22, 29]. In a sequential design, the data are collected in multiple stages. In theory, the clustering parameters could be estimated as the data are collected and the design could then be changed to accommodate the observed clustering values. However, the variance of clustering parameters is high in small samples. Further, sequential designs are logistically more challenging. Future research should evaluate such sequential designs in simulation and in practice. Users should keep in mind that overestimating clustering parameters at the design phase is always more conservative and adds to the cost of the survey.

In practice, to select an lqas survey design, the user first must decide whether srs is feasible and practical. If srs is used, survey design can proceed using a standard binomial model and the simple steps described in the Introduction. If srs is not used, two-stage cluster sampling is a commonly used practical alternative. In some situations, the cluster sampling design can be ignored when small within-cluster sample sizes are used [17, 19–21] or when clustering is known to be negligible [22]. Survey design is much simpler when the cluster sampling design is ignored, because the user can act as though the data are a simple random sample and use standard lqas procedures for design; that is, the user does not need to specify a clustering model or specify the clustering amount (i.e. *ρ* or *σ*). When the cluster sampling design cannot be ignored, choice between the cluster lqas methods (Pezzoli, Hedt, and Hund) is not straightforward. Historically, *ρ*, not *σ*, is fixed as a function of *p* in design calculations [16, 25]. Uniting the methodology for lqas surveys with other m&e estimation surveys may help de-mystify lqas. The binomial-scaled model is not used in the statistics or survey sampling literature, possibly due to the discrete nature of this distribution. However, based on the empirical calculations in this article, choice between the Pezzoli, Hedt, and Hund methods should depend more upon the anticipated clustering patterns in the study population than the distributional specification (though prior information on clustering is likely quite limited). Hence, further research should attempt to characterize clustering patterns in specific applications and provide suggestions for best-practice cluster lqas designs on a setting-specific basis. We only considered data examples in the context of vaccination coverage cluster LQAS surveys in this paper, but the validity of different clustering parameterizatons should be evaluated in other contexts, for instance nutrition.

Cluster sampling in lqas has also been recently discussed in the context of Large Country lqas (lc-lqas) [18, 30, 31]. These lc-lqas surveys select sas using cluster sampling with the goals of estimating prevalence over a large geographic region and classifying indicators at the sa-level. The lc-lqas surveys are distinct from cluster lqas surveys discussed in this paper which sample clusters *within* sas, rather than sampling sas.

Future work could explore accommodating uncertainty in *ρ* or *σ* at the design phase. In the data examples in this paper, both *ρ* and *σ* vary with *p*, complicating selection of a cluster lqas method. All three methods could be modified to allow *σ* or *ρ* to vary as a function of *p*, though this modification would introduce an additional design parameter into the lqas procedure. Future work should investigate the merits of considering designs with different clustering parameters corresponding to *p*_*l*_ and *p*_*u*_ or exploring different clustering parameterizations [25]. Additionally, all of the cluster surveys discussed in this paper rely on using a self-weighting sampling design. Future work should explore how to extend lqas designs for unequal probability weights.

## Appendix

Assume we have a model where *p*_*j*_ ∼ *F* with *E*(*p*_*j*_) = *p* and *Var*(*p*_*j*_) = *σ* ^2^ and *X*_*j*_ ∼ *Binomial*(*m*, *p*_*j*_). Let ${\hat{p}}_{j}=X_{j}/m$. To estimate *σ* ^2^ from summary statistics, note that:

$$\begin{array}{c} Var\left({\hat{p}}_{j}\right)=E\left\{Var\left({\hat{p}}_{j}|p_{j}\right)\right\}+Var\left\{E\left({\hat{p}}_{j}|p_{j}\right)\right\}=E\left\{\frac{p_{j}\left(1-p_{j}\right)}{m}\right\}+\sigma^{2}. \end{array}$$

Additionally, $E\{p_{j}(1-p_{j})\}=E(p_{j})-E(p_{j}^{2})=p(1-p)-\sigma^{2}.$

Then, $Var({\hat{p}}_{j})=\frac{p(1-p)-\sigma^{2}}{m}+\sigma^{2}.$

Solving for *σ* ^2^, we have that $\sigma^{2}=\frac{mVar({\hat{p}}_{j})-p(1-p)}{m-1}.$

Plugging in the empirical variance of the ${\hat{p}}_{j}$ s for $Var({\hat{p}}_{j})$ and $\hat{p}$ for *p*, the final estimator is:

$$\begin{array}{c} {\hat{\sigma }}^{2}=\frac{ms^{2}-\hat{p}\left(1-\hat{p}\right)}{m-1}. \end{array}$$

This estimator is unbiased for *σ* ^2^ as *m*, *k*, or *mk* gets large (i.e. $Var(\hat{p})\to 0$ or *m* → ∞), since $E({\hat{\sigma }}^{2})=\sigma^{2}+\frac{Var(\hat{p})}{m-1}.$

Note that ${\hat{\sigma }}^{2}<0$ when $s^{2}<\hat{p}(1-\hat{p})/m$ (within-cluster variance > between cluster variance). When estimates of *σ* are negative, we use ${\hat{\sigma }}^{2}=0$.
